# Optimal Thawing of Cryopreserved Peripheral Blood Mononuclear Cells for Use in High-Throughput Human Immune Monitoring Studies 

**DOI:** 10.3390/cells1030313

**Published:** 2012-07-25

**Authors:** Hari Ramachandran, Jessica Laux, Ioana Moldovan, Richard Caspell, Paul V. Lehmann, Ramu A. Subbramanian

**Affiliations:** Cellular Technology Limited, Shaker Heights, OH 44122, USA; Email: Hari.Ramachandran@stemcell.com (H.M.); Jessica.Laux@immunospot.com (J.L.); Ioana.Moldovan@immunospot.com (I.M.); Richard.Caspell@immunospot.com (R.C.); Paul.Lehmann@immunnospot.com (P.V.L.)

**Keywords:** ELISPOT, PBMC, T cells, cryopreservation, DMSO

## Abstract

Cryopreserved peripheral blood mononuclear cells (PBMC) constitute an important component of immune monitoring studies as they allow for efficient batch- testing of samples as well as for the validation and extension of original studies in the future. In this study, we systematically test the permutations of PBMC thawing practices commonly employed in the field and identify conditions that are high and low risk for the viability of PBMC and their functionality in downstream ELISPOT assays. The study identifies the addition of ice-chilled washing media to thawed cells at the same temperature as being a high risk practice, as it yields significantly lower viability and functionality of recovered PBMC when compared to warming the cryovials to 37 °C and adding a warm washing medium. We found thawed PBMC in cryovials could be kept up to 30 minutes at 37 °C in the presence of DMSO before commencement of washing, which surprisingly identifies exposure to DMSO as a low risk step during the thawing process. This latter finding is of considerable practical relevance since it permits batch-thawing of PBMC in high-throughput immune monitoring environments.

## 1. Introduction

Many established immune assays in wide use today require timely and coordinated access to assay instrumentation and highly trained technical personnel to handle sample runs and data acquisition. In the context of complex clinical studies that entail immune monitoring, coupling the use of freshly isolated peripheral blood mononuclear cells (PBMC) with downstream bioassays and sample runs presents significant logistical challenges. Increased potential for variation in data acquired over protracted periods of time, often from multiple study centers, is an important concern in designing clinical studies. Importantly, sole reliance on fresh cells also limits future access to samples for additional biological studies or validation. Cryopreservation of biological samples, when optimally performed, overcomes many of these challenges; we have previously demonstrated that cryopreserved T cells, of both CD4 and CD8 lineages, maintain full functionality in cytokine ELISPOT assays following thawing [1]. Strong evidence suggests cryopreservation is a reliable and convenient alternative to the use of fresh PBMC, resulting in its widespread use in both basic and clinical studies [2,3,4,5,6,7,8,9]. 

The osmotic, temperature, and solute changes that the PBMC undergo during cryopreservation and thawing could significantly affect the viability and functionality of the recovered cells. We have previously shown that the use of pre-chilled freezing media used in traditional cryopreservation methods [10,11] significantly impairs the viability and functionality of frozen cells while use of room temperature freezing results in optimal viability and functionality of PBMC [1]. While general consensus suggests gradual freezing leads to optimal viability of cryopreserved cells by minimizing the formation of ice crystals both within and outside the cells undergoing cryopreservation [12], systematic comparison of thawing conditions of cryopreserved PBMC is currently missing. 

Significant variation exists in the PBMC thawing methodology utilized in the immune assay field. While PBMC are generally thawed by placing cryovials in 37 °C water baths, presently there is no consensus protocol specifying whether the washing medium should be added when the last ice crystals are visible or whether the cells should be warmed first to 37 °C, and if so, how long they can stay at 37 °C. Protocols also differ in the temperature of the washing medium used (warm *vs.* cold) and the speed at which the washing medium is added [1,13]; alternatively, some studies do not specify these details [14,15]. Cox and coworkers document significant variation in viability of identical PBMC samples when 11 independent laboratories were allowed to follow their own standard operating procedures (SOP) for PBMC thawing [16]; specifically, the study observed a median viability of 86% with a wide range (24.8% to 100%).

In the current study, we systematically compare the effect of temperature and rapidity of thawing on the viability and functionality of cryopreserved PBMC and the extent to which the duration of exposure to DMSO affects PBMC viability and functionality. The study also addresses if CD4 and CD8 cell-driven responses are differentially affected by the different conditions during the thawing procedure.

## 2. Results

### 2.1. The Effect of Temperature and Speed of the Wash Medium Addition during Thawing on the Viability and Functionality of Cryopreserved PBMC

Lab-specific variations are common in how cryopreserved PBMC are thawed and such variations have the potential to affect the performance of lymphocytes in downstream immune assays [1,13,14,17]. We formally assessed how the rapidity and temperature at which thawing is performed affects the viability and functionality of the cryopreserved PBMC. Specifically, cells were subjected to either “cold-” or “warm” processing. For “cold processing”, cryovials were thawed until the last ice crystals were visible, and ice-chilled media was added immediately to the ice-cold cells. For “warm processing”, the cryovials were incubated in a 37 °C bead bath for 10 minutes which raised the temperature of the cells in the cryovial to 37 °C. For both conditions, we also compared the impact of adding the media rapidly or slowly, as specified in the Experimental Section. Thus, a total of four thawing conditions were tested: (1) slow addition of 37 °C warm media to cells at 37 °C; (2) rapid addition of 37 °C media to cells at 37 °C; (3) slow addition of ice-chilled media to ice-cold cells, and; (4) rapid addition of ice-chilled media to ice-cold cells. PBMC derived from 7 donors were subjected in parallel to these thawing conditions and were evaluated for viability in initial experiments. 

**Figure 1:**
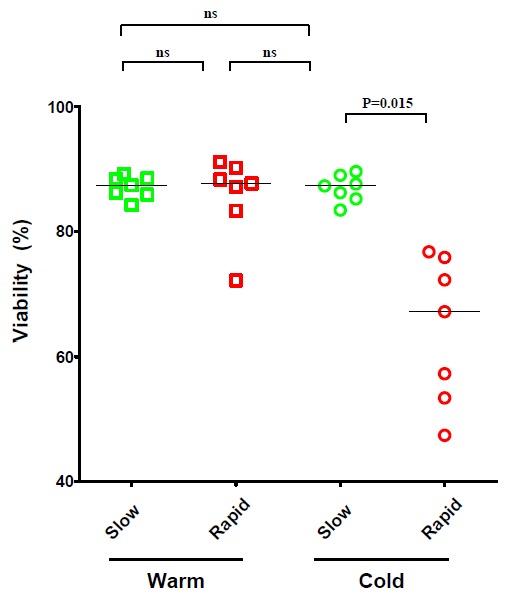
Viability of cryopreserved peripheral blood mononuclear cells (PBMC) from seven subjects following thawing under warm or cold processing conditions. Warm wash medium was added to warm cells, and cold wash medium to cold cells, at a rate of 1 mL/5 seconds (slow) or in a single stream <5 seconds (fast), as specified in the Experimental Section. The viability of the cells was assessed by acrydin orange / ethidium bromide staining. Comparison of viability at various thawing conditions were performed by nonparametric Wilcoxon signed rank test with two-tailed p values ≤ 0.05 being considered significant; (ns) not significant.

Data depicted in Figure 1 shows, of the four conditions tested, adding ice-chilled media rapidly to ice-cold cells strongly reduced the recovery of viable PBMC. Slow addition of ice-chilled media overcame this drop in viability. Therefore, in spite of the use of the cold media, its slow addition overcomes the drop in viability of the cells. Warm media, regardless of the speed of addition, provided comparably high levels of cell viability (Figure 1).

For further evaluation of thawing conditions, we chose to compare the least optimal condition (adding ice-chilled media rapidly to ice-cold cells) with an optimal warm condition (37 °C media added slowly to PBMC that have been warmed to 37 °C). The prior experiment was repeated using cryopreserved PBMC derived from ten additional donors. 

**Figure 2:**
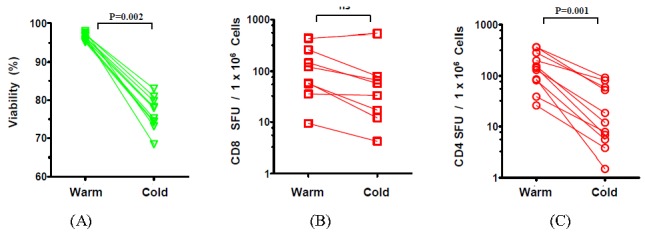
Viability and functionality of cryopreserved PBMC from 10 subjects following thawing under warm or cold processing conditions. (**A**) Post-thaw viability of PBMC under both conditions was assessed as described in Figure 1. (**B**) CD8 ELISPOT responses were measured using the CEF peptide pool that comprised of defined Class I peptides from CMV, EBV, and influenza viruses [18,19] as the antigen to stimulate the PBMC that were thawed according to the “warm-” or “cold processing protocol. (**C**). CD4 ELISPOT responses: mumps and mosquito antigen were used to stimulate CD4 cells. Statistical comparisons were performedwith Wilcoxon signed rank test with two-tailed p values ≤0.05 being considered significant.

In agreement with the original finding, the data from these ten donors also showed that the use of cold media significantly reduced (*p* = 0.002, Wilcoxon signed rank test) the viability of thawed PBMC (Figure 2A). 

Next, we sought to establish if the different thawing conditions affected CD8 and CD4 cell functionality. To evaluate CD8 cell functionality, we used the CEF peptide pool comprising of defined Class I peptides from CMV, EBV, and influenza viruses that elicit recall CD8 responses in most donors [18,19] that elicited varying magnitudes of CEF responses among our donors. CEF-specific responses were assayed in 8 out of 10 donors due to paucity of PBMC from two donors (8 data points). To similarly test anamnestic CD4 cell responses, we first tested a variety of protein antigens including those derived from Mumps, Mosquito, Dust Mite, Candida, and PPD among the study subjects. In our cohort of 10 donors, most represented CD4 responses were toward mumps antigens (5 donors) and mosquito (6 donors) with lower number of donors responding to the other antigens. Only one donor had significant levels of candida antigen-specific responses. For further analysis of CD4 functionality, we concatenated responses from the most represented mosquito and mumps antigens (11 data points). Figure 2B shows while CD8 cells showed a tendency toward reduced functionality under “cold processing” conditions, this difference was not statistically significant. In stark contrast, CD4-driven responses directed against mumps and mosquito antigens were significantly impaired under “cold processing” conditions (Figure 2C). Specifically, when compared to “warm processing”, “cold processing” resulted in a 1.98-fold diminution in the median CD8 responses and 12.05-fold diminution in median CD4 responses. 

### 2.2. The Effect of the Number of Washing Steps during Thawing on the Viability and Functionality of Cryopreserved PBMC

Wash steps during the PBMC thawing procedure aim to remove the cryopreservant DMSO which is toxic for lymphoid cells [20,21] and reduces their functionality in bioassays [22]. While increasing the number of washes will dilute out the residual DMSO from thawed PBMC, it also increases the procedural time and leads to cell loss. Potentially, the prolonged processing may also affect the viability and functionality of the cells. To directly address if increasing the number of washing steps affects the viability and/or functionality of recovered PBMC, we thawed the cells under “warm processing” conditions and assessed viability and functionality of the cells following either 1 or 2 washes. 

**Figure 3:**
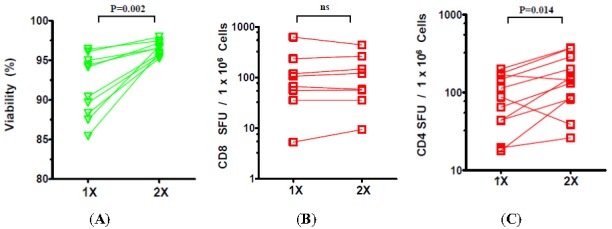
Effect of number of washes during the PBMC thaw procedure on CD8 and CD4 ELISPOT responses post-thaw. Viability (**A**), CD8 cell function (**B**) and CD4 cell function (**C**) were assessed after one or two washes, as specified in the Experimental Section utilizing antigens used in Figure 2.

Data shown in Figure 3 suggests two washes significantly increase the viability of the recovered PBMC when compared to samples that underwent a single wash (Figure 3A). While functionality of the CD8 compartment (CEF peptide pool responses) was not significantly affected by the number of washes performed (Figure 3B) the functionality of CD4 cells was significantly impaired when the second wash was not implemented (Figure 3C). 

### 2.3. The Effect of Prolonged Post-Thaw DMSO Exposure at 37 °C on the Viability and Functionality of Cryopreserved PBMC

Because prolonged exposure to DMSO is known to be toxic to varying lineages of cells [20,21,23] we sought to assess how long PBMC can be exposed to the DMSO once thawed and warmed to 37 °C before their viability and/or functionality is significantly affected. Following removal from liquid Nitrogen and thawing, the PBMC containing cryovials were warmed to 37 °C in the bead bath for 10 minutes, and kept at 37 °C for additional 0, 30 or 60 minutes before the cells were diluted and washed two times with warm media, counted and tested in ELISPOT assays. 

**Figure 4:**
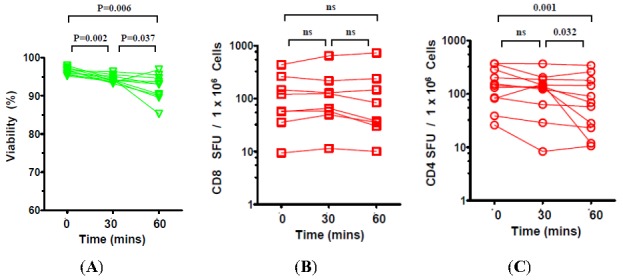
Effect of post-thaw DMSO exposure at 37 °C on the viability and functionality of cryopreserved PBMC. Cryovials were kept in a bead bath at 37 °C for 10 minutes, followed by an additional 0, 30, and 60 minutes as specified on the X axis. Viability (**A**), CD8 cell function (**B**) and CD4 cell function (**C**) was assessed as specified in the Legend to Figure 2.

While incubating the cryovials at 37 °C caused a significant decline in viability of thawed PBMC (Figure 4A), this diminution was minimal throughout the entire 60 minute incubation period in absolute terms. Specifically, while we observed a median 96.6% viability when DMSO was washed immediately post-thaw (zero time point), viability was at 94.2% when the washes were performed at 30 minutes (2.4% drop from zero time point) and at 93.0% when washes were performed at 60minutes (further 1.2% drop). Thus, the total decline in viability over a 60 minute period was only 3.6%. 

When assessed for T cell functionality, we observed no significant reduction in the CD8 functionality over the entire 60 min incubation in the presence of DMSO (Figure 4B). Similarly, we found no significant reduction in CD4 functionality during the initial 30 minutes (median SFU of 144.6 at zero time point *vs.* 137.6 SFU per 10^6^ cells). However, further exposure to DMSO for up to 60 minutes (Figure 4C) resulted in significant reduction in CD4 cell functionality detected in downstream ELISPOT assays (median of 68.9 SFU / 10^6^ cells). These data suggests a safe window of up to 30 minutes may exist where DMSO exposure does not significantly affect either CD4 or CD8 functionality in ELISPOT assays with the CD8 positive cells being even more refractive to longer incubation in the presence of DMSO. 

## 3. Discussion

The use of cryopreserved PBMC to assess cellular immunity has found wide use in human immune monitoring studies [5,24,25,26,27]. Specifically, complex immune studies in diverse fields such as cancer biology and vaccinology have increasingly become more feasible given our ability to effectively cryopreserve PBMC and assess them immunologically at later convenient periods [5,25,26,28]. Access to fully functional cryopreserved PBMC allows for effective deployment of skilled labor and resources in batched immune assays, and the ability to minimize variability seen in assays preformed in multi-center trials and among samples collected over protracted periods of time. 

We previously reported that optimally cryopreserved PBMC can be thawed and utilized in downstream immunoassays without loss of CD4 or CD8 T cell functionality [1]. This was the first study to systematically assess the effect of cryopreservation on T cell functionality using ELISPOT: a key finding from this study was that the addition of chilled washing medium to chilled cells can be highly detrimental to PBMC viability (Figure 1 in that publication). ELISPOT provides a reliable quantitative approach to assess functionality of diverse immune cell types including T cells of both CD4 and CD8 lineages [29,30,31]. We and others have utilized ELISPOT effectively to assess immunogenicity of both candidate- and licensed vaccines [5,26,32]. 

The current study makes a number of practical observations related to the thawing of PBMC that can prove invaluable in high-throughput immune monitoring environments. Data from this study suggests that rapid removal of DMSO following thawing of PBMC may not be essential. If PBMC are to be washed immediately of DMSO following thawing, as recommended by classic protocols, this would in practice require thawing of cryovials in small staggered batches, a cumbersome practice that increases the complexity and time required to complete protocols in high-throughput environments. Data from this study suggests functionality of both CD4 and CD8 cells are retained during a 30 minute incubation period with DMSO with CD4 responses declining upon longer incubation in the presence of the cryoprotectant (Figure 4). As no decline in CD8 functionality occurs over a 60 minute period, studies focusing on CD8 responses may have a longer window period for sample processing. These data are in agreement with a recent study that found even longer periods of tolerance to DMSO exposure (up to 2 hours) though the authors utilized freshly isolated PBMC and not the more stressed, cryopreserved cells [33]. 

The rapidity and temperature of DMSO removal during PBMC thawing is also subject to significant lab-specific variation [1,13,14,17]. Our systematic comparison of conditions that vary these parameters suggests that “warm processing” (adding warm washing medium to warm cells) provides better viability of cells when compared to “cold processing” (adding chilled media to cold cells) as shown in Figure 1. When working cold, the cells are sensitive to the speed at which the washing medium is added, suggesting that adding the medium slowly represents a gentler treatment (Figure 1). The PBMC we utilized were from healthy donors and were frozen under ideal conditions that we have previously described [1]. It is conceivable, therefore, that adding warm wash medium slowly may also prove beneficial when more fragile cells or PBMC acquired during disease states or sub-optimally frozen cells are utilized. Therefore, our data provides a basis for recommending slow addition of warm media to the warm cells to dilute out DMSO during PBMC thawing. 

The data presented here suggests two washes are required for the detection of optimal CD4 responses though CD8 responses were unaffected by increasing the number of washes (Figure 3). For testing CD8 cells, therefore even a single wash suffices. This observation may have relevance to the peptides used in immune assays which are frequently dissolved in DMSO to ensure solubility in the aqueous test medium. DMSO concentrations of <0.1% are generally thought to be permissible in T cell immune assays [34]. Though the actual number of viable cells recovered following freeze-thaw is bound to vary among cryopreserved PBMC derived from different subjects, a rough estimate of final DMSO concentrations present during the ELISPOT assay can be made. As the cells were cryopreserved in 10% DMSO (in 1 mL), after the initial dilution with 10mL wash media, the DMSO concentration can be estimated to have dropped to 1% in the pelleted cell volume (~50 µL). The resuspension of the pellet in an estimated 2 to 3 mL media (1: 40 or 1:60 dilution) and its further dilution during addition to antigen containing wells (1:2) will result in DMSO concentrations significantly below the acceptable 0.1% threshold. The fact the CD4 functionality is affected more than CD8 functionality even at this range of DMSO suggests that DMSO could potentially impact antigen processing and presentation of the protein antigens more so than the minimal peptides that stimulate CD8 cells. Further studies are warranted to test this hypothesis. 

Taken together, the data presented here suggests a more conservative approach is needed for optimal immune monitoring of CD4+ cells as significant functional impairment of these T cells occurs if adequate washing and or/rapid processing at optimal temperatures are not performed. It is conceivable cryopreservation affects, functionally or numerically, one or more cell subsets including antigen presenting cells or the metabolic pathways involved in costimulation that differentially affect CD4 responses while sparing CD8 responses. It is also conceivable that specific memory subsets within low frequency antigen-specific CD4+ T cells become less viable following freeze-thaw. To establish why CD4 cells are more sensitive to suboptimal thawing conditions is beyond the scope of this study. However, this study clearly establishes the ideal thawing conditions that result in full functionality of both CD4 and CD8 cells and can thus significantly advance our ability to successfully execute high- throughput immune monitoring studies. 

## 4. Experimental Section

### 4.1. Thawing and Handling of Cryopreserved PBMC and Antigens

Cryopreserved PBMC were selected from a characterized library (CTL-CP1) available through Cellular Technologies Limited (CTL, Shaker Heights, Ohio). PBMC cryovials were transferred from liquid Nitrogen vapor phase to dry ice in styrofoam containers and were thawed by placing them for 10 minutes in a CTL Bead Bath™ (CTL-BB-001). Cryovials were inverted twice to resuspend the PBMC and the cells from a single cryovial (10 million cells) were transferred into a 15 mL Falcon tube utilizing a wide-bore 2 mL pipette. We found the use of nominal buffers such as PBS during washing resulted in suboptimal cell viability when compared to the use of CTL Anti-Aggregate Wash™ Medium (CTL-AA-005) which was specifically formulated to ensure optimal viability and functionality of thawed PBMC. This formulation includes supplementary nutrients and Benzonase (a DNAase shown to reduce clumping of cells during the thawing procedure). Cryovials were rinsed by adding 1mL CTL Anti-Aggregate Wash™ Medium to recover residual cells. For the “slow” wash, additional 8 mL CTL Anti-Aggregate Wash™ Medium was added at a rate of 1 mL/5 seconds. For “fast wash, the 8 mL wash medium was added in a shot. The CTL Anti-Aggregate Wash™ Medium was added either at 37 °C (“warm”) or ice-chilled (“cold”) as specified in the figures. An aliquot of diluted PBMC was counted by fluorescence microscopy using acridine orange and ethidium bromide to stain live and dead cells respectively. PBMC were washed by centrifugation at 330g for 10 min either once or twice in 10 mL of 37 °C or ice-chilled media. Cells were resuspended at a final concentration of 3 × 10^6^ PBMC/mL in CTL-Test™ Medium (CTLT-005). 

### 4.2. Human Interferon-γ ELISPOT Assay

Human Interferon-γ ImmunoSpot kits (CTL-HIFNG-1/5M) were acquired from C.T.L and the ELISPOT assay was performed according to the manufacturer’s recommendations. The membranes were not pre-wetted with ethanol as it is not required nor recommended for this kit. The following antigens were added first at appropriate concentrations in 100 µL/per well: CEF peptide pool (CTL-CEF-002; 2 µg/mL); mosquito antigen (Greer Labs, Lenoir, NC; 100 µg/mL) and mumps antigen (BioWhittaker, Walkersville, MD, USA, Lot# IV0094, 1:80 dilution). Plates were maintained at 37 °C in a CO_2_ incubator until the cells were ready for plating. Thawed PBMC were adjusted in CTL Test Medium (CTLT-005) at 3 million PBMC/mL and the cells were plated at 100 µL/well (300,000 cells/well) using wide-bore pipette tips. Plates were gently tapped on each side to ensure even distribution of the cells and incubated for 24 hours at 37 °C in a CO_2_ incubator. Following completion of the ELISPOT protocol according to kit recommendations, the plates were air dried in a laminar flow hood prior to analysis. 

ELISPOT plates were scanned and analyzed using an ImmunoSpot S6 Core Reader (C.T.L). Spot Forming Units (SFU) were automatically calculated by the ImmunoSpot software for each antigen stimulation condition and the media (negative) control using the SmartCount™ and Autogate™ functions [35]. Data are presented as mean SFU per 10^6^ PBMC induced by the specified antigen minus the SFU count in the negative control. In all experiments, the negative control was less than 10 SFU per 10^6^ PBMC.

## 5. Conclusions

We can make the following recommendations for PBMC thawing, based on this systematic study: thaw PBMC by incubating Cryovials minimally for 10 minutes and maximally for 30 minutes at 37 °C. Add 37 °C medium slowly, at a rate of 1 mL/ 5 seconds. Wash cells twice. We also recommend the use of a 37 °C bead bath rather than a water bath for thawing the cells to decrease the risk of contamination.
